# Evolutionary and network analysis of virus sequences from infants infected with an Australian recombinant strain of human parechovirus type 3

**DOI:** 10.1038/s41598-017-04145-2

**Published:** 2017-06-20

**Authors:** Soren Alexandersen, Tiffanie M. Nelson, Jason Hodge, Julian Druce

**Affiliations:** 1 Geelong Centre for Emerging Infectious Diseases, Geelong, Victoria, 3220 Australia; 2 Deakin University, School of Medicine, Geelong, Victoria, 3220 Australia; 3 Barwon Health, University Hospital Geelong, Geelong, Victoria, 3220 Australia; 4 Victorian Infectious Diseases Reference Laboratory (VIDRL), Doherty Institute, Melbourne, Victoria, 3000 Australia

## Abstract

We present the near complete virus genome sequences with phylogenetic and network analyses of potential transmission networks of a total of 18 Australian cases of human parechovirus type 3 (HPeV3) infection in infants in the period from 2012–2015. Overall the results support our previous finding that the Australian outbreak strain/lineage is a result of a major recombination event that took place between March 2012 and November 2013 followed by further virus evolution and possibly recombination. While the nonstructural coding region of unknown provenance appears to evolve significantly both at the nucleotide and amino acid level, the capsid encoding region derived from the Yamagata 2011 lineage of HPeV3 appears to be very stable, particularly at the amino acid level. The phylogenetic and network analyses performed support a temporal evolution from the first Australian recombinant virus sequence from November 2013 to March/April 2014, onto the 2015 outbreak. The 2015 outbreak samples fall into two separate clusters with a possible common ancestor between March/April 2014 and September 2015, with each cluster further evolving in the period from September to November/December 2015.

## Introduction

The picornaviruses are small, single-stranded positive sense RNA viruses causing disease in animals and humans and include the human parechoviruses (HPeV). Like other picornaviruses, HPeV evolve rapidly due to nucleotide substitutions and recombination events, generating strains associated with changed infectivity, virulence or host range. The first detected outbreak of HPeV type 3 (HPeV3) in infants in Australia occurred in and around Sydney in New South Wales in late 2013, with subsequent infection in other parts of Australia in the first part of 2014. A second outbreak occurred across Victoria and Southern Australia in the second half of 2015. As described previously^1^ this latter outbreak included the first HPeV3 cases in infants seen at University Hospital Geelong and was shown to be caused by a novel recombinant strain of HPeV type 3^1^. We now extend our previous study to include the near complete virus genome sequences and the phylogenetic and transmission network analyses of 12 of the Geelong 2015 cases and another 6 Australian cases from 2012–2015 for a total of 18 HPeV3 infant cases in Australia. Thus, our study provides valuable, near complete genome HPeV3 sequences and explores the potential value of phylogenetic and network analyses for better understanding HPeV3 transmission and evolution over time.

HPeV type 1 (HPeV1) and 2 were initially classified as echoviruses 22 and 23 in the genus *Enterovirus*, but reclassified in 1998/99 into their own genus based on nucleotide and biological features including a lack of host cell protein synthesis shut-off during replication^2–6^. The RNA genome of HPeV is approximately 7300-7400 nucleotides with 5′ and 3′ untranslated regions (UTR) at each end of a single long open reading frame (ORF)^7–9^. The single ORF encodes a polyprotein including the P1 structural/capsid and the P2 and P3 nonstructural proteins. The capsid proteins of HPeV consist of only 3 proteins, VP0, VP3 and VP1, as VP0, in contrast to some other picornaviruses, is not proteolytically cleaved into VP4 and VP2^7, 10^. Some of the HPeV types have an arginine-glycine-aspartic acid (RGD) motif near the carboxy-terminus of VP1, thought to be the receptor-binding site likely binding to host cell integrins^11, 12^. However, this RGD motif is not present in HPeV3, HPeV7-16, the related rodent/zoonotic virus Ljungan virus or in some strains within HPeV types normally encoding the RGD motif, for example for HPeV4^7, 13–16^. Like other picornaviruses, recombination of HPeV often involves breakpoints at the very end of VP1 and thus may remove or add the RGD motif, or alternatively occur downstream in the nonstructural coding region^7, 17–21^.

HPeV infections are prevalent in humans and most often cause only asymptomatic/subclinical or mild gastrointestinal and/or respiratory disease. More severe disease including central nervous system (CNS) dysfunction may be seen in particular in infants and one relatively large study reported mortality among young infants as high as 6%^22, 23^. HPeV3 appears to cause more severe disease in young infants compared to the other HPeV types and is the most common type identified in cerebrospinal fluid (CSF) samples from young infants with CNS infection and/or sepsis-like presentation^22^. Evaluation by cranial magnetic resonance imaging (MRI) or ultrasound of a group of 10 infants with HPeV3 CNS infection showed abnormal periventricular white matter in all infants and for a number of the infants, clinical follow-up revealed sequelae including cerebral palsy, epilepsy, learning disabilities, and visual impairment^24, 25^. Further cases like this including reports of autopsy cases of two infants that died with active HPeV3 infection and severe CNS disease are referenced in ref. 22. In regard to pathogenesis, it appears that HPeV3 may target vascular smooth muscle cells in the leptomeninges, and in the lung, and thus that clinical symptoms and severe CNS tissue damage may be caused by vascular changes^22^.

HPeV3 was first isolated in 1999 and reported in the literature in 2004 and subsequent surveillance suggests a two to three-year cycle between outbreaks^11, 23, 26–33^. The reason for this is not known and as these viruses are easily transmitted by the oral-faecal and respiratory routes^30^, it may be related to exposure and immunity within families e.g. outbreaks occurring when there is both an older sibling lacking prior HPeV3 exposure plus waning or no maternally derived HPeV3 passive immunity in the young infant^30, 34^. Studies from Japan have indicated that antibodies to HPeV3 are only present in 15% of children from 7 months to 1 year of age, but that seropositivity increases to 45% in 2–3 year old and to 85% in 4–6 year old children^11, 35^. Due to these factors, virus testing results and virus sequences available for analyses are likely to only represent a minority of HPeV3 infections. Therefore, transmission network analyses will help elucidate part of a much larger transmission network, i.e. will represent sequences sampled from severe disease in young infants among a much larger population of infected older children and adults with mild or no clinical disease and thus not sampled and tested.

Molecular clock analyses have estimated that the parechoviruses have existed for approximately 400 years with HPeV3 and HPeV7 diverging approximately 150 years ago^36^. However, it appears that more recent strains have spread worldwide only within the last 20–30 years^28, 37^, and during this time, may have evolved into being more virulent. One such example may be the Yamagata 2011 lineage of HPeV3 shown to cause severe disease in young children and myalgia in adults^38–40^. We have previously shown that the first reported outbreaks of HPeV3 in infants in Australia in 2013/14 and 2015, respectively, were caused by a novel recombinant HPeV3 with the capsid encoding region closely related to the Yamagata 2011 lineage, while the nonstructural encoding region derived from an as yet unknown/unpublished HPeV(s)^1^.

We now present the near complete virus genome sequences with phylogenetic and network analyses of potential transmission from a total of 12 of the Geelong 2015 cases as well as from another 6 positive Australian cases. These include an early Australian sample from 2012 obtained by retrospective testing, samples from Sydney from 2013 and 2015 in addition to two samples from Adelaide and one from Darwin from 2014 making a total of 18 cases of HPeV3 in infants in Australia. Overall the results support our previous finding^1^ that the major recombination event took place between March 2012 and November 2013 and we now show evidence of further temporal virus evolution and possibly recombination. While the nonstructural coding region appears to be evolving both at the nucleotide and amino acid level, the capsid encoding region appears to be very stable, particularly at the amino acid level. This apparent stability of the virus capsid may indicate that development of a vaccine or antibody treatment to avoid severe disease in infants may be feasible. It should be noted that the ancestor of the nonstructural coding region of the Australian recombinant HPeV3 is still not known and further sequencing and publishing/depositing of results from cases worldwide should be a priority to further the understanding of generation, evolution, spread and possibly even host range of new/recombinant strains of HPeV3.

## Results and Discussion

Next generation sequencing (NGS) was performed on RNA extracted from the clinical samples (Table 1) as well as on available virus isolates. The coverage and quality of the sequences obtained varied somewhat between samples and those with a lower coverage or quality were included in multiple runs. A total of 5 NGS runs were performed to obtain sufficient data to assemble high quality consensus sequences for all samples included. Sample coverage varied from approximately 83000 mapped reads with an average coverage of 2400 for sample CS-HP-16018 (which is a sample from 2012 and not the novel recombinant Australian outbreak virus^1^, thus not fully covered by our Ampliseq panels), to 2.3 million mapped reads with an average coverage of 67000 for sample CS-HP-16012. Apart from samples CS-HP-16018 mentioned above and CS-HP-16003 for which we only had very limited sample material left from our previous study, samples averaged 0.5–1.5 million reads and an average coverage of 14000–50000. This allowed us to assemble high quality near complete virus genome consensus sequences from each sample apart from sample CS-HP-16018, where high quality sequences were obtained for the first approximately 5000 nucleotides only. For that sequence we have chosen to only report and include in our analyses the capsid encoding region from nucleotide 701–3013. All sequences generated have been deposited in GenBank under accession numbers KY556659-KY556676.

**Table 1:** Sample and patient information.

GCEID identification^a^	Age in weeks^b^	Sample type^c^	Short sample designation^d^	Time^e^	Home location^f^
CS-HP-16001	<1	CSF	CSF01	November 2015	20 km south east of Geelong
CS-HP-16003	7	CSF	CSF03	December 2015	20 km south east of Geelong
CS-HP-16004	12	CSF	CSF04	September 2015	20 km east of Geelong
		Faecal	FEC04		
CS-HP-16005	8	CSF	CSF05	October 2015	South Geelong
CS-HP-16006	4	CSF	CSF06	October 2015	5 km south of Geelong
		Faecal	FEC06		
		Nasal	NAS06		
CS-HP-16007	11	CSF	CSF07	October 2015	5 km east of Geelong
CS-HP-16008	3	CSF	CSF08	September 2015	Central Geelong
CS-HP-16010	9	Faecal	FEC10	September 2015	South Geelong
CS-HP-16012	4	Faecal	FEC12	September 2015	South Geelong
CS-HP-16014	15	Nasal	NAS14	October 2015	20 km north east of Geelong
CS-HP-16016	11	Nasal	NAS16	November 2015	350 km north east of Geelong
CS-HP-16017	11	Faecal	FEC17	September 2015	Central Geelong
CS-HP-16018	6	Nasal	NAS18	March 2012	20 km east of Geelong
CS-HP-16019	1	CSF	CSF19	November 2013	Sydney^g^
CS-HP-16020	7	Faecal	FEC20	September 2015	100 km north of Sydney^g^
CS-HP-16021	7	Faecal	FEC21	March 2014	Adelaide^h^
CS-HP-16022	19	Faecal	FEC22	April 2014	Darwin^i^
CS-HP-16023	11	Faecal	FEC23	April 2014	Adelaide^h^

The Table displays GCEID sample identification^a^, age of the affected infant when samples taken^b^, the sample type including cerebrospinal fluid (CSF)^c^, short sample designation^d^, time of sample taken^e^, and approximate home location of infant^f^. ^g^Sydney is located approximately 800 km north east of Geelong. ^h^Adelaide is located approximately 630 km north west of Geelong. ^i^Darwin is located approximately 3100 km north of Geelong. Each of the shown cases tested positive for HPeV by reverse-transcription real-time polymerase chain reaction (rRT-PCR), conducted at the Victorian Infectious Disease Reference Laboratory (VIDRL). The Table only includes data for samples for which we had sufficient sample material to produce high quality near full-length HPeV3 sequences.

We first compared sequences obtained from different sample types from the same patient (CSF and faecal samples obtained from CS-HP-16004 and CSF, faecal and nasal samples from CS-HP-16006; Table 1). The sequences obtained from the different samples collected from the individual patient were in 100% agreement. These results support the robustness and accuracy of the NGS sequences and moreover, allowed us to focus on a single sequence from each case in the phylogenetic analyses. As a second step, we compared the sequences obtained from virus isolates (see Materials and Methods) to the sequences obtained from the corresponding clinical sample. For 4 out of the 6 virus isolates available, the obtained sequence of 7334 nucleotides were in 100% agreement with that obtained from the clinical sample, while sample CS-HP-16018 and CS-HP-16019 each had a single non-coding change between the clinical sample and the corresponding virus isolate. For sample CS-HP-16018 the single non-coding nucleotide change was located in the capsid coding region and for CS-HP-16019 it was located in the 5′-UTR. The comparison between clinical samples and corresponding virus isolates served two purposes. First, it further supports the very high accuracy of the NGS generated sequences indicated by comparing different sample types mentioned above. Second, it suggests that the obtained virus isolates do not appear to have been selected/adapted during the cell culture process and may accurately reflect *in vivo* virus, which is useful information to have if a virus isolate is used for animal trials or *in vitro* studies.

As a first step in our analyses of the sequences obtained, we screened for recombinants among the sequenced viruses using GARD (genetic algorithm recombination detection) analysis. We discovered in our previous study that the Australian HPeV3 outbreak virus is a recombinant, with the sequence up to approximately nucleotide 3115 derived from a Yamagata 2011-like virus, whereas the downstream sequence is derived from recombination(s) with an as yet unknown/unpublished HPeV/s^1^. The analysis done here did not include CS-HP-16018 as we did not have the near full-length sequence and moreover, have already demonstrated that the virus in this sample is not the novel recombinant virus as in the other Australian HPeV3 samples sequenced, but rather consistent/similar to a full, i.e. not further recombined, Yamagata 2011 lineage virus^1^. Interestingly, although only indirectly related to our analyses presented here and to our knowledge not reported in the literature, the Yamagata 2011 lineage viruses reported^38–40^ and deposited in GenBank contain what appear to be two different clusters most likely derived by recombination at approximately nucleotide 5000 (equivalent to nucleotide 4300 in the deposited Yamagata 2011 lineage viruses as they only included the coding regions). These two clusters are represented by GenBank Accession number AB759207 in one of the two clusters and AB759204 and AB759205 in another. We inadvertently based our initial NGS panel on AB759207^1^ and this particular panel coincidentally resulted in high quality sequence for sample CS-HP-16018 up to approximately nucleotide 5000, consistent with this sample, from March 2012, being of full Yamagata 2011 lineage, but really matched most closely to the AB759204/AB759205 cluster/recombinant and not the AB759207 cluster/recombinant. Nevertheless, based on BLAST comparison to all sequences available in the NCBI databases, the Yamagata 2011 lineage(s) of viruses are the closest related to our Australian HPeV3 outbreak viruses in the region up to approximately nucleotide 3100 (the capsid encoding region) and consequently are included in our initial phylogenetic analysis. In any event, GARD analysis of the full sequence and the coding region from nucleotide 701 to 7231 of our Australian HPeV3 virus sequences, (excluding CS-HP-16018 as mentioned above), indicated possible sites of recombination at nucleotide 997 and nucleotide 3025 or 3550. The level of significance only reached p = 0.01–0.05 and likely reflects the impact of slight differences in evolutionary rates among the regions derived from previous recombination event(s)^1^. The sequences found to have differences in topologies of segments in the GARD analysis were samples CS-HP-16003, 16006, 16007, 16010, 16014, 16017 and 16020, the potential importance of this is addressed later in the discussion. Nevertheless, in our phylogenetic analyses described below, we looked at the regions both separately and as full-length sequences to see an effect, if any, of a prior site of recombination. Sequences were divided into the capsid encoding region (nucleotides 701–3013); the nonstructural proteins encoding region (nucleotides 3014–7231); the full protein encoding region/ORF (nucleotides 701–7231); or finally, the full-length sequences from nucleotide 1–7334. The dataset from nucleotides 701–3013 included either the 17 sequences from the Australian samples containing the novel recombinant strain or another set with 21 sequences containing these 17 Australian recombinant virus samples and in addition sample CS-HP-16018, an Australian sample from 2012 with a fully Yamagata 2011 lineage virus, as well as the 3 closest related Yamagata 2011 virus sequences obtained from NCBI (i.e. AB759204, AB759205 and AB759207). For the remaining datasets we only included the 17 Australian samples containing the novel recombinant HPeV3, as the region downstream of the capsid coding region is likely to be derived from a previous recombination event/s and consequently will have a different evolutionary history than the fully Yamagata 2011 lineage viruses^1^.

The transition/transversion bias, nonsynonymous (dN)/synonymous (dS) nucleotide substitutions per site ratio (dN/dS), indication of positive or negative selection of sites and number of variable nucleotide and amino acid sites in the datasets are shown in Table 2. The range of the calculated transition/transversion bias in the datasets was from 7.85–9.68 using the maximum likelihood (ML) method and from 9.1–18.2 using the maximum composite likelihood (MCL) method depending on the dataset. This shows that the variation among the sequences is heavily biased towards transitions, in particular in the capsid encoding region that also had the lowest dN/dS ratio of 0.06 compared to the nonstructural coding region and the full ORF with a ratio of 0.16 and 0.13, respectively. This is consistent with a strong indication of negative/purifying selection in the capsid coding region and some indication of positive selection at a few sites in the nonstructural coding region (Table 2). The sites of potentially positive selection included codon 826 in the 2 A coding region where samples from 2013 and 2014 had a lysine (CS-HP-16019, 16022, 16023) or an arginine (CS-HP-16021), while the Sydney sample from 2015 (CS-HP-16020) had a methionine and all 12 Geelong 2015 samples had an isoleucine. The other two potentially positive selected codons were codon 1502 in the 3 C coding region having a serine in most of the samples, but a glycine in samples CS-HP-16003, 16006, 16007 and 16014 and finally codon 2067 in the 3D coding region having an alanine in most samples, but a valine in samples CS-HP-16001, 16003, 16006, 16007, 16014 and 16017. The significance of these changes, if any, is currently unknown.

**Table 2:** Information about the sequence datasets analysed.

**Sample set**	GARD	SLAC p0.05	FEL p0.01	FEL p0.05	IFEL p0.05	REL 100	dN/dS	Tra/Trv-ML	Tra/Trv-MCL	NT diffs	NT%	AA diffs	AA%
**701-3013 incl sample18 & Yamagata**	nil	nil	neg cod 713	neg 7 codons	neg cod 713	neg 55 codons	0.061	9.17	9.11	64/2313	2.77	9/771	1.17
**701-3013 17 samples**	nil	nil	nil	neg codon 53, 175 & 511	nil	neg 25 codons	0.060	9.68	18.2	29/2313	1.25	4/771	0.52
**3014-7234 A 17 samples**	nil	nil	nil	nil	nil	POS codon 55, 731&1296 REM add 771 to codon	0.160	8.33	9.69	117/4218	2.77	28/1406	1.99
**701-7234 A 17 samples**	nt 3550 p0.01	neg codon 53 & 175	neg codon 53 & 175	neg codon 53, 175 & 511	neg codon 53 & 175	POS codon 826, 1502 & 2067	0.132	8.7	10.62	146/6531	2.24	32/2177	1.47
**1-7334 A 17 samples**	nt 997 p0.01 & nt 3025 p0.05	NA	NA	NA	NA	NA	NA	7.85	9.52	154/7334	2.1	NA	NA
5′**-UTR 1-700**	ND	NA	NA	NA	NA	NA	NA	8.01	2.84	8/700	1.14	NA	NA
**3′UTR 7232-7334**	ND	NA	NA	NA	NA	NA	NA	NA	NA	0/103	0	NA	NA

The sample sets and results of various types of analyses are shown; see Materials and Methods and Results and Discussion sections for details. Tra/Trv is the transition/transversion bias. Nucleotide (NT) and amino acid (AA) sites with differences within each dataset are also shown.

**Figure 1:**
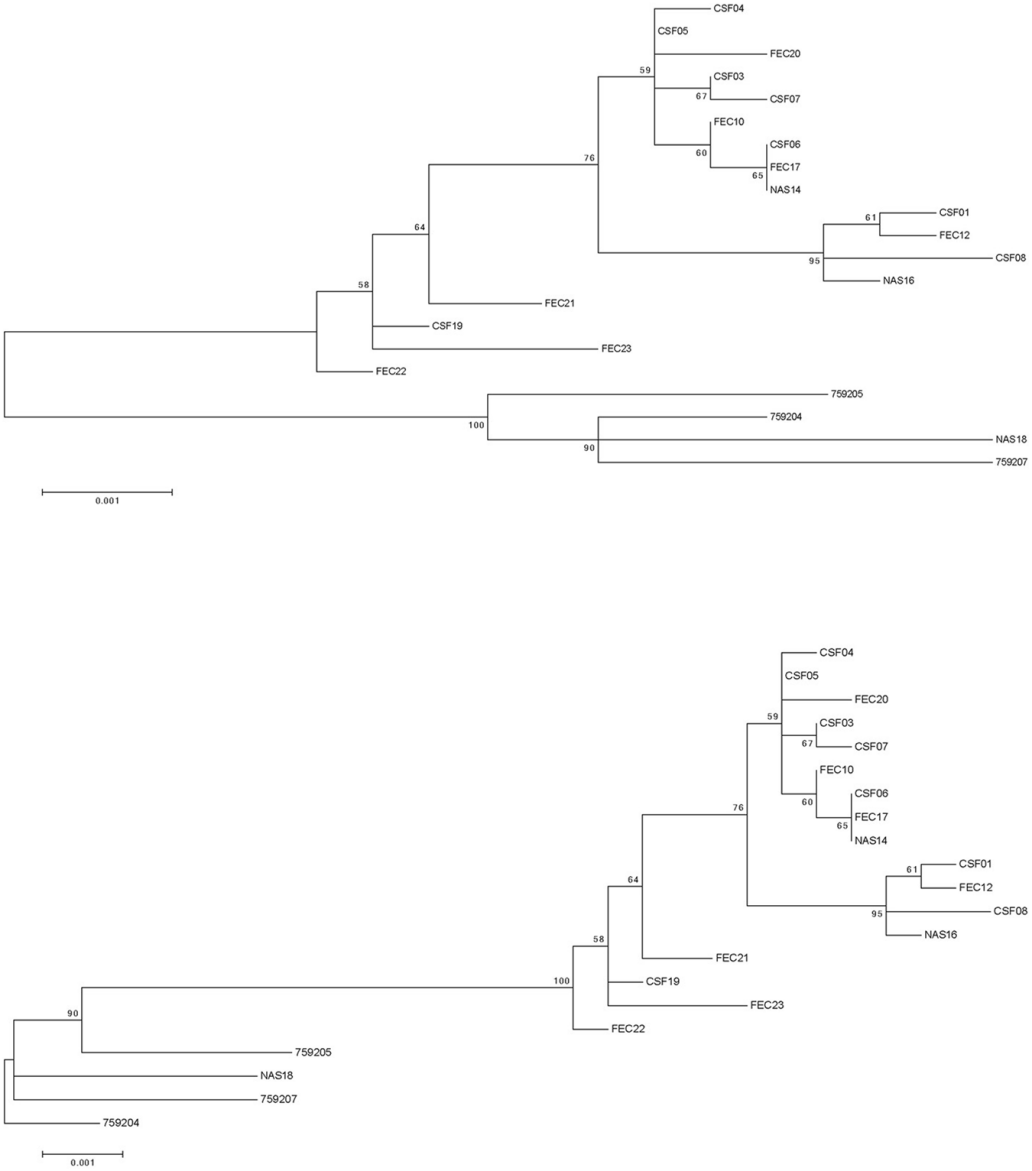
Maximum Likelihood (ML) phylogenetic tree based on the capsid encoding region from nucleotide 701–3013 of the 18 Australian HPeV3 sequences together with the closest related Yamagata 2011 virus sequences. Sequences were aligned using Clustal W and phylogenetic analysis was conducted using MEGA 6 and the Maximum Likelihood (ML) method based on the Tamura-Nei model^51^. The tree with the highest log likelihood is shown. Bootstrap test involved 1000 replicates to determine reliability of the inferred tree. The numbers at nodes represent bootstrap values. Branch lengths are scaled according to the numbers of nucleotide substitutions per site. Details of the samples are given in Table 1 and in the text. Samples 759204, 759205 and 759207 are the closest related Yamagata 2011 lineage virus sequences and taken from NCBI with the corresponding accession numbers: AB759204, AB759205, AB759207. Based on information from AB759206 (HPeV3 Yamagata 2011 lineage^39^), the structural/capsid coding region is from nucleotide 701–3013 and the nonstructural protein coding region from 3014–7231 and inferred from that the 5′-UTR spans nucleotide 1-700 and the 3′-UTR from 7232–7334 including 12 As of the poly-A tail. A) Midpoint rooted tree and B) rooted on the closest related non-Australian sequence, AB759204.

**Figure 2:**
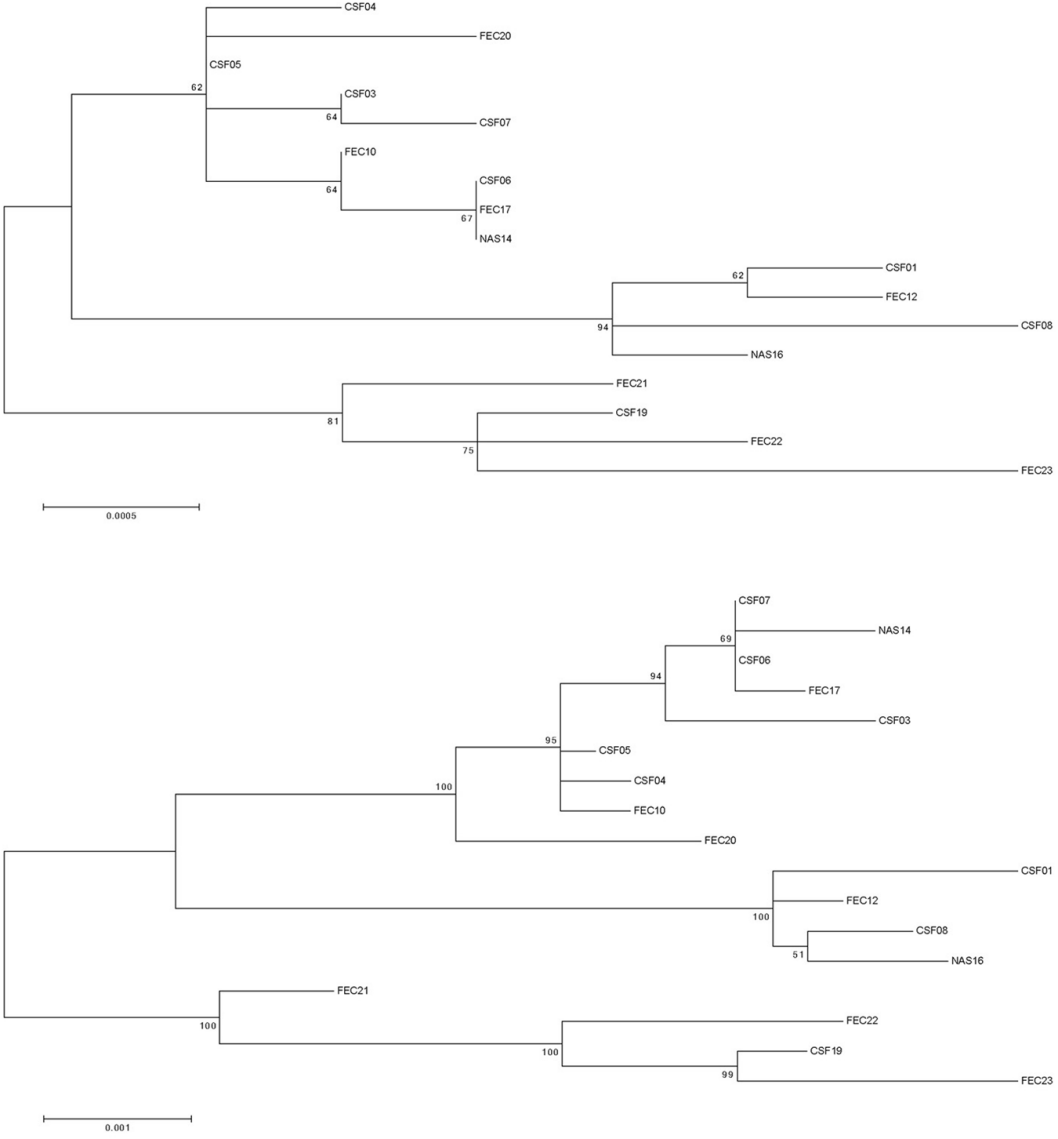
Maximum Likelihood (ML) phylogenetic trees based on either the capsid or nonstructural proteins encoding region of the 17 Australian 2013–2015 HPeV3 sequences. Sequences were aligned using Clustal W and phylogenetic analysis was conducted using MEGA version 6 and the Maximum Likelihood (ML) method. The tree with the highest log likelihood is shown. Bootstrap test involved 1000 replicates to determine reliability of the inferred tree. The numbers at nodes represent bootstrap values. Branch lengths are scaled according to the numbers of nucleotide substitutions per site. (A) Midpoint rooted tree based on the capsid encoding region from nucleotide 701–3013. (B) Midpoint rooted tree based on the nonstructural proteins encoding region from nucleotide 3014–7231.

**Figure 3:**
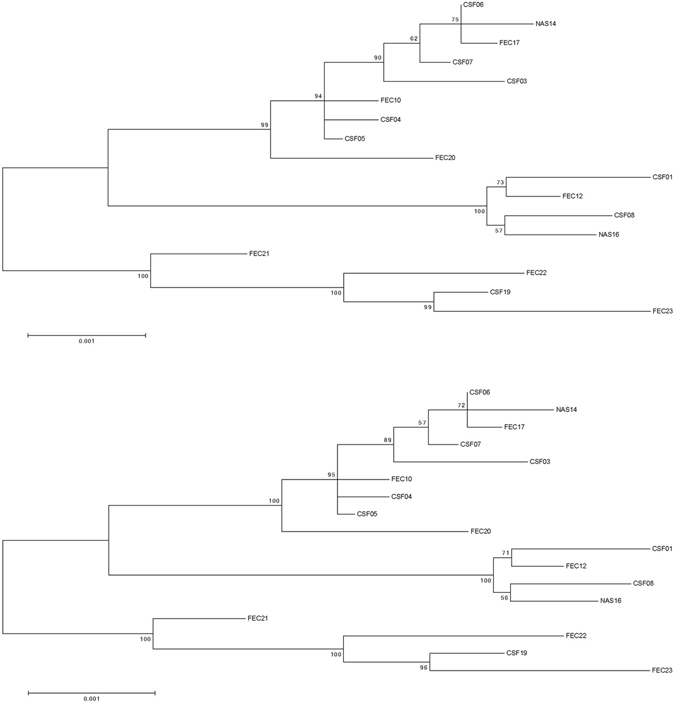
Maximum Likelihood (ML) phylogenetic trees based on the either the full open reading frame from nucleotide 701–7231 or the full length sequence from nucleotide 1–7334 of the 17 Australian 2013–2015 HPeV3 sequences. Sequences were aligned using Clustal W and phylogenetic analysis was conducted using MEGA version 6 and the Maximum Likelihood (ML) method. The tree with the highest log likelihood is shown. Bootstrap test involved 1000 replicates to determine reliability of the inferred tree. The numbers at nodes represent bootstrap values. Branch lengths are scaled according to the numbers of nucleotide substitutions per site. (A) Midpoint rooted tree based on the full open reading frame from nucleotide 701–7231. (B) Midpoint rooted tree based on the full length sequence from nucleotide 1–7334.

The next step was to do phylogenetic inference of the sequence datasets by Maximum Likelihood (ML) phylogenetic analyses using the MEGA 6 software. Phylogenetic trees are shown in Figs 1–3.

Overall the ML phylogenetic analyses show that the capsid region of the Australian recombinant HPeV3 virus sequences are related to each other and more distantly to the Yamagata 2011 virus sequences. The Australian virus sequence from 2012 (sample NAS18/CS-HP-16018) has the closest relation to the Yamagata 2011 virus while the other Australian virus sequences from 2013/14 are more related to the Yamagata 2011 sequences than the Australian virus sequences from 2015. This latter group partitioned into two clusters; cluster 1 with CSF samples CS-HP-16003, 16004, 16005, 16006, 16007, faecal samples CS-HP-16010, 16017, 16020 and nasal sample CS-HP-16014 and a cluster 2 with CSF samples CS-HP-16001, 16008, faecal sample CS-HP-16012 and nasal sample CS-HP-16016 falling separately (Fig. 1A and B). It should be noted that this clustering may have been caused by another recombination event as the samples in cluster 1, with the exception of CS-HP-16004 and 16005, all were identified by the GARD analysis as having potential recombination sites or other cause of incongruent evolution as compared to the other samples included here. Interestingly, the analysis indicated that the Australian 2013/14/15 recombinant outbreak strain/lineage was more closely related to AB759204 Yamagata 2011 than to the “non-recombinant” 2012 Australian virus (nasal sample CS-HP-16018). The midpoint rooted tree obtained for the 17 Australian recombinant virus sequences for the capsid region (Fig. 2A) is very similar to that for the nonstructural coding region (Fig. 2B) although the variability for the nonstructural coding region is somewhat higher (Fig. 2 and Table 2). A similar picture and relationship is also evident in the trees for the full ORF (Fig. 3A) and for the full sequence from nucleotide 1–7334 (Fig. 3B). Thus, although the different regions appeared to be under different evolutionary pressures, inferences as shown by the clustering and topology of the trees generated, were relatively robust. Overall, this analysis indicated that the 17 Australian recombinant outbreak virus sequences had the 2013/14 virus sequences clustering together while the 2015 virus sequences fell into two separate discrete clusters as mentioned above.

**Figure 4:**
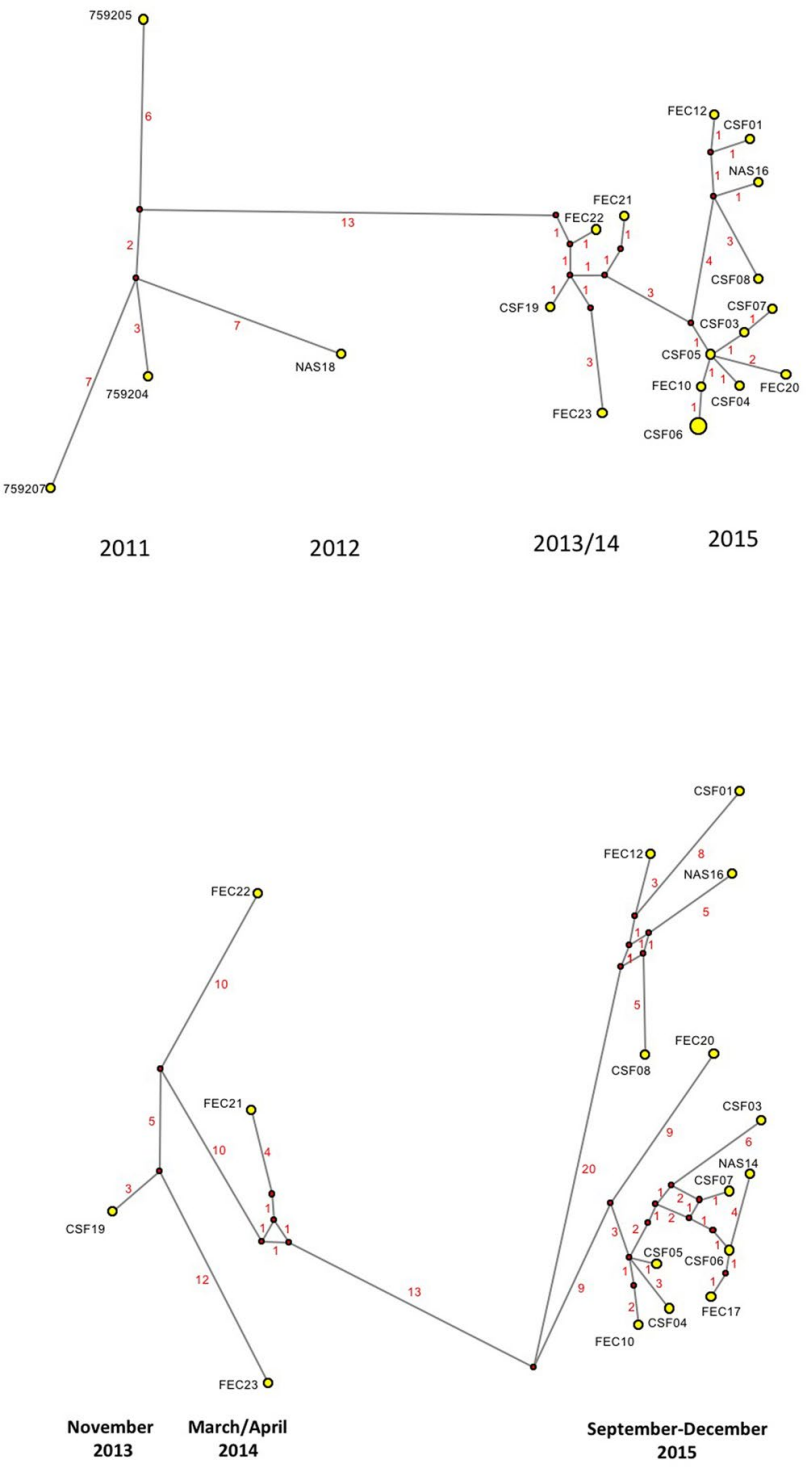
Median-joining phylogenetic network of the analysed HPeV3 sequences. The networks shown include all of the most parsimonious trees linking the sequences. Each unique sequence is represented by a circle showing the identity and frequency in the dataset. Branch length is proportional to the number of nucleotide differences and the number of nucleotide differences between nodes are shown in red. (A) Network based on the capsid encoding region from nucleotide 701–3013 and including all 18 Australian sequences as well as the 3 selected Yamagata 2011 lineage virus sequences as indicated in Fig. 1. The larger circle labeled CSF06 also includes samples NAS14 and FEC17 as the sequences of these 3 samples/cases were identical for the capsid encoding region. Year of samples obtained shown at the bottom. (B) Network analysis based on the full open reading frame from nucleotide 701–7231 and only including the 17 Australian virus sequences from 2013–2015, i.e. the novel Australian recombinant HPeV3. Month and year of samples obtained shown at the bottom. For more details on samples see Table 1.

Phylogenetic transmission network construction was then done using the program Network v5. It should be emphasized that our virus sequences all came from severely ill infants and likely only represent a fraction of infected individuals. Consequently, the aim was not to look at direct transmission between these cases, but rather to look for an indication of a potential transmission network likely containing a large number of unsampled infections among older children and adults (likely to only show mild or no clinical symptoms and therefore not tested for HPeV). Similar to the ML phylogenetic analysis shown above, we did this for the individual regions, for the full ORF and for the full sequence. Similar to what is mentioned above, we found the potential transmission networks generated to be very similar and again suggesting that the closest ancestor for the capsid region of the recombinant Australian 2013/14/15 virus sequences was AB759204 of the Yamagata 2011 virus lineage. Interestingly, while the Australian 2012 “non-recombinant” virus sequence (sample NAS18/CS-HP-16018) clearly was closely related to the Yamagata 2011 virus sequences, it may not be the direct ancestor of the subsequent Australian outbreaks that may be caused by separate introduction(s) (Fig. 4A) [21 sequences, capsid only]. As the apparent transmission networks for the regions, the full ORF and the full sequence were very similar, and moreover the non-coding regions only had a total of 8 variable sites (Table 2) we have chosen to just show the network inferred from the full ORF consisting of 6531 nucleotides and 2177 codons (Fig. 4B) [17 sequences]. The potential transmission network shown in Fig. 4B corresponds well to the ML tree in Fig. 3A. Furthermore, it also supports the temporal evolution from the first Australian recombinant virus sequence from November 2013 to March/April 2014 onto the 2015 outbreak. Also evident are the two 2015 clusters mentioned previously with a likely common ancestor between March/April 2014 and September 2015 and each cluster further evolving in the period from September to November/December 2015. The only discrepancy in this apparent temporal network is faecal sample FEC17 (CS-HP-16017) that is positioned after samples from October yet was taken in September 2015. However, as HPeV is very stable in the environment, we anticipate that this infection would include many unsampled infected individuals. In this outbreak, this discrepancy could most likely be explained by case CS-HP-16017 being infected relatively early in September 2015, possibly by someone at the end of a long unsampled network, or alternatively, may be explained by some cases in that cluster (i.e. CSF samples CS-HP-16006 and CS-HP-16007, both sampled in October) derived from cases that have been infected from virus excreted into the environment already in September. Another difference between the ML tree in Fig. 3A and the transmission network in Fig. 4B involves sample CSF19 (CS-HP-16019) which although sampled in November 2013 is not consistently basal to the Australian samples from early 2014 (FEC21, 22 and 23). The reason for this is unknown, but may at least in part be explained by the fact that CSF19 appears to be more closely related to sample FEC23 than to the other samples from 2014 and moreover, that the trees get somewhat skewed by the fact that FEC22 is more closely related to the included Yamagata 2011 lineage viruses than to CSF19. However, considering that these particular samples (CSF19 and FEC21, 22 and 23) represent a very few samples from affected infants separated by many hundreds of kilometres (Table 1), speculation on exact causes is not possible apart from noting the fact that although both collected from cases in Adelaide and only separated in time by one month, samples FEC21 and FEC23 appear to represent two slightly different clusters somewhat similar to what is observed for the Geelong 2015 cases (Fig. 4A and B).

In addition to the temporal aspect, we also attempted to look at the apparent transmission network from a spatial perspective using the approximate home location of each case (Table 2 **)**. Not unexpectedly, considering the anticipated large number of unsampled infected individuals and the likely wide travel network involved, in particular in relation to the holiday season up to December, we could not detect any spatial connection between either the 2015 clusters, or the earlier Australian virus sequences. However, for the 2015 virus sequences it is interesting to note that those with the highest number of nucleotide differences and located most distant in the transmission networks are the faecal sample CS-HP-16020 taken in Sydney and the CSF CS-HP-16001, CS-HP-16003, CS-HP-16008 and nasal CS-HP-16014, CS-HP-16016 samples taken from infants treated at University Hospital Geelong which, except for CSF sample CS-HP-16008, have home addresses located outside or even at some distance from Geelong (Table 1 and Fig. 4B).

We have here reported near complete virus genome sequences from 17 samples and a partial sequence of one sample essentially doubling the availability of near complete HPeV3 genome sequences in the NCBI databases. Furthermore, we show that consensus sequences obtained from different sample types or directly from clinical sample as compared to virus isolate are essentially identical. Overall our results support our previous finding that the major recombination event leading to the generation of the Australian 2013–2015 outbreak virus took place between March 2012 and November 2013 possibly followed by additional recombination events contributing to the evolution of seperate clusters. While the nonstructural coding region derived from an as yet unknown HPeV appears to be evolving both at the nucleotide and amino acid level and may still be evolving/adapting to its full replicative potential in this recombinant virus setting, the capsid encoding region appears to be very stable, in particular at the amino acid level. As we have noted previously, the stability of the virus capsid indicates that development of a vaccine or antibody treatment to avoid severe disease in infants may be feasible. However, the ancestor of the nonstructural coding region of the Australian recombinant HPeV3 is still not known and we encourage further sequencing and publishing/depositing of results from cases worldwide in order to further the understanding of generation, evolution, spread and possibly even host range of such new/recombinant strains of HPeV3.

## Materials and Methods

### Clinical specimens and virus isolates

An outbreak of HPeV3 was identified in infants in the Geelong area of Victoria, Australia during August-December 2015 and a total of 16 cases were identified at the University Hospital Geelong as described previously^1^. For the studies described here we had available a total of 15 samples from 12 of these cases and also 3 stored clinical samples from the Victorian Infectious Disease Reference Laboratory (VIDRL) collection described previously^1^. An additional 3 positive samples selected from VIDRL’s stored collection of HPeV3 positive samples from Australia provided a total of 21 clinical samples from 18 cases of HPeV3 in infants in Australia; see Table 1 for details.

Virus isolates from the following 6 samples were also available for analysis: CS-HP-16006 faecal and nasal swab samples, CS-HP-16004 faecal sample, CS-HP-16018 nasal sample, CS-HP-16019 CSF sample and CS-HP-16020 faecal sample. These virus samples were cultured on monolayers of Vero cells as described previously^1^.

All samples were stored at −80C and RNA extracted and converted into cDNA as described previously^1^.

The studies described here were performed in accordance with relevant guidelines and regulations and was provided with ethical exemption by the Barwon Health Human Research Ethics Committee.

### Next generation sequencing (NGS) of HPeV-positive samples

As described previously^1^, we initially constructed a composite reference genome from related HPeV3 sequences available in NCBI (National Center for Biotechnology Information) and used this initial reference (here called composite reference 1, see ref. 1 to design a custom Ion AmpliSeq Panel (here referred to as panel 1) for use with Ion Torrent S5 System (Thermo Fisher Scientific, Vic. Australia). Based on initial NGS results and additional Sanger sequencing (described previously^1^), we subsequently updated this initial reference sequence (updated reference available as GenBank accession number KY020128) and used that to design an updated Ion Ampliseq Panel, referred to as panel 2. Based on additional NGS runs, we further updated the reference sequence and designed a third Ion Ampliseq Panel, here referred to as panel 3. Details of panel 2 and 3 can be seen online (Supplementary Table S1).

Extracted RNA converted to cDNA from all samples were PCR-amplified with the Ion AmpliSeq Library Kit 2.0 and Ion AmpliSeq Panel 1, 2 or 3. The panels comprise 37 or 38 overlapping primer sets split into 2 pools per sample and amplified with the 5X Ion AmpliSeq HiFi Master Mix. The details of our NGS protocol has been described in detail previously^1^ and for the results described here we only made minor adjustments including raising the number of initial PCR cycles from 30 to 35 cycles and then leaving out the post-amplification step and finally, we used the Ion Library TaqMan™ Quantitation Kit to quantify the amplified libraries. Libraries were then pooled prior to loading onto Ion 530 Chips and loading into the Ion Chef Instrument. Following template preparation, the chips were run on the Ion Torrent S5 System following company protocols. NGS and associated reactions were performed at the Geelong Centre for Emerging Infectious Diseases (GCEID), Geelong, Victoria, Australia. For the NGS results described here we did a total of 5 Ion Torrent S5 runs with a total of 6 Ion 530 Chips generating a total of approximately 25 billion nucleotides.

### Next generation sequence analyses

Sequences obtained from the Ion Torrent S5 were analysed based on each sample having a unique barcode. Raw sequences were analysed using the Ion Reporter Software contained within the Ion S5 Instrument using either of the 3 reference genomes or various HPeV reference genomes from NCBI. Obtained reads were then visualised and analysed using the Integrative Genomics Viewer (IGV)^41^. IGV calculates the consensus sequence for the NGS results for each sample as described by Cavener^42^; if the frequency of a single nucleotide at a specific position is greater than 50% and greater than twice the number of the second most frequent nucleotide it is assigned as the consensus nucleotide or if the sum of the frequencies of two nucleotides is greater than 75% (but neither meet the criteria for a single nucleotide assignment) they are assigned as co-consensus nucleotides. If no single nucleotide or pair of nucleotides meets the criteria, IGV assign an ‘N’. In the few occasions where our consensus sequences contained co-consensus nucleotides at a given position, we manually inspected the results and assigned a single nucleotide when a given nucleotide was present in 60% or more of the reads. The final assembled virus genome sequences were based on the NGS results indicated above, but also included nucleotides 1–29 and 7292–7334 (the very 5′- and 3′end sequences). These did not derive directly from the NGS sequencing, but are assumed based on: (i) the high efficiency of the end-primers used; (ii) comparison to other closely related sequences, and (iii) the fact that the rest of the 5′- and 3′-UTR sequences obtained were highly conserved as indicated in the results.

### Phylogenetic analyses and transmission network construction

Screening for recombinants among the sequenced viruses were done using GARD (genetic algorithm for recombination detection) analyses^43, 44^ and the HyPhy package^45, 46^ available on the Datamonkey webserver and using the HKY85 substitution model as suggested by using the automatic Model Selection Tool available on the site. The GARD technique identifies evidence for recombination breakpoints by searching multiple sequence alignments for phylogenetic incongruences.

To understand phylogenetic relationships among the HPeV3 sequences, they were compared to the NCBI database using nucleotide BLAST (Basic Local Alignment Search Tool)^47, 48^ and aligned using Clustal-W^49^. Maximum Likelihood phylogenetic analyses were conducted using the MEGA 6 software^50^ and the Tamura-Nei model^51^ (identical results were obtained using the HKY85 and the HKY + G models, data not shown). The robustness of different nodes was assessed by bootstrap analysis using 1000 replicates. To understand the chains of transmission between obtained samples, sequences were used to construct phylogenetic (transmission) networks using the Median Joining method implemented in the program Network v5 (http//www.fluxus-engineering.com) developed to reconstruct all possible shortest, least complex phylogenetic trees (all maximum parsimony) and depict this as a network. The parameter epsilon was set at the same value as the weight of characters used to calculate the genetic distances (weight value = 10) as described previously^52^. As the virus sequences available for this analysis most likely only represent a minority proportion of HPeV3 infections occurring in the total transmission network, i.e. only represent sequences sampled from severe disease in young infants among a much larger population of infected older children and adults with mild or no clinical disease and thus not sampled, connections in the transmission network are not an indication of direct contact transmission between individual cases. Rather, it should be viewed as cases linked by chains of transmission that include an unknown number of mild or undiagnosed infections, yet still able to indicate temporal evolution and chains of transmission.

### Analysis of selection pressure

Transition/transversion bias was estimated using Maximum Likelihood (ML) and Maximum Composite Likelihood (MCL) analysis available in MEGA 6. Site-specific selection pressures were measured as nonsynonymous (dN) -synonymous (dS) nucleotide substitutions per site and estimated using the single-likelihood ancestor counting (SLAC), fixed-effects likelihood (FEL), internal fixed-effects likelihood (IFEL), and random effects likelihood (REL) methods available at the Datamonkey online version of the HyPhy package. All analyses utilized the HKY85 nucleotide substitution model, which was tested as the best fitting model for the data sets, and employed input Neighbor Joining phylogenetic trees. A cut-off p-value to classify a site as positively or negatively selected was set at 0.01 or 0.05 for SLAC, FEL, and IFEL methods and the cut-off value for the Bayes factor in the REL method was set at 100 to reflect a positive or negative selection at a given site as described previously^52^.

### Data Availability

All sequences generated have been deposited in GenBank under accession numbers KY556659-KY556676. Other datasets generated or analysed during the current study are available from the corresponding author on reasonable request.

## Electronic supplementary material

Supplementary Table S1
