# Supplementary material for: Evolutionary and network analysis of virus sequences from infants infected with an Australian recombinant strain of human parechovirus type 3
Source: Sci Rep. 2017 Jun 20;7:3861. doi: 10.1038/s41598-017-04145-2 (PMC5478645; doi:10.1038/s41598-017-04145-2)
Supplement: Supplementary file 1 — Supplementary Table S1 [file 41598_2017_4145_MOESM1_ESM.doc]

**Supplementary materials**

**Evolutionary and network analysis of virus sequences from infants infected with an Australian recombinant strain of human parechovirus type 3**

**Running title**

Evolutionary and network analysis of an Australian recombinant strain of human parechovirus type 3

**Authors**

Soren Alexandersen^1,2,3*^, Tiffanie M. Nelson^1,2^, Jason Hodge^1,3^, Julian Druce^4^

^1^Geelong Center for Emerging Infectious Diseases, Geelong, Victoria 3220, Australia

^2^Deakin University, School of Medicine, Geelong, Victoria 3220, Australia

^3^Barwon Health, University Hospital Geelong, Geelong, Victoria 3220, Australia

^4^Victorian Infectious Diseases Reference Laboratory (VIDRL), Doherty Institute, Melbourne, Victoria 3000, Australia

**^*^Corresponding author** e-mail soren.alexandersen@deakin.edu.au

**Table S1. Ion Ampliseq Panels Custom Primer Sequence Pairs**

| Panel | Amplicon | Forward primer | Reverse primer | Amplicon start | Insert start | Insert stop | Amplicon stop |
| --- | --- | --- | --- | --- | --- | --- | --- |
| Panel 2 (0 9207) | AMPL1678933 | GGGTCTCCTAGAGAGCTTGG | GTACAACACACTTTAAGCGTCTGAAC | 9 | 29 | 191 | 217 |
|  | AMPL1678934 | CTGGCCGAAGGCAACTAGCAAT | AGAGGCATCTGTTACCAGATGTG | 159 | 181 | 407 | 430 |
|  | AMPL1678935 | ATTTTGCTGCAAAGCATCCCATT | CCAACTCACCTAGATAGAGGTAGTTAGC | 374 | 397 | 605 | 633 |
|  | AMPL1678936 | CGTAGGTAACAAGTGACACTATGGA | CACACTAGACAAGGAGAGAGTATCCA | 570 | 595 | 743 | 769 |
|  | AMPL1678937 | GAGTCTATCAAGGATTTAGTCAATGTTGC | CTTGCACTACATCTTTATTCTCTGGTTG | 704 | 733 | 883 | 911 |
|  | AMPL1678938 | TCCAATTTACTTGGACCAAACAGTTTTG | CCACAGTGGTTATTTTGTACTGTCTAGAAT | 845 | 873 | 1088 | 1118 |
|  | AMPL1678939 | TAAGCTTATAAGATTGGACACAGCTTCATG | CTTCCTGCAGTTCCTTGGTTGA | 1048 | 1078 | 1223 | 1245 |
|  | AMPL1678940 | TAAGGTGTGGCTTTCATTTCCAAGT | GGTACTGAAAGAGGTGTCCAAACA | 1188 | 1213 | 1437 | 1461 |
|  | AMPL1678941 | CGATTCGTCTGATCTAGGGCAA | GTTCCAGGCCCTTCTGCTATATC | 1405 | 1427 | 1654 | 1677 |
|  | AMPL1678942 | GACTGCTTCAACTAAATACAAATGGACAC | GCCCAATCAAAGTACCCATATTTGTCTATA | 1615 | 1644 | 1854 | 1884 |
|  | AMPL1678943 | GTTATGTCAGACAACACTACACCTTCA | CTCCAACTGAGTATCATGAGTACAGTTG | 1817 | 1844 | 2058 | 2086 |
|  | AMPL1678944 | GTACTTTCAATAGAGGAAGGCTTAGGATG | ACTTCAACTTGAAATAAACCCAATTGGTG | 2019 | 2048 | 2188 | 2217 |
|  | AMPL1678945 | CCATATTCCTTCTCTACTTGGATGAGGA | TCATTTGGTGATATGGACTGTTTGCA | 2150 | 2178 | 2398 | 2424 |
|  | AMPL1678946 | AGATCCATTGTGCATTGAGGACA | GCCATGACCTTCTTTTGGAAATGT | 2365 | 2388 | 2614 | 2638 |
|  | AMPL1678947 | TCATGATTTTAACAATGGAGACACATGGA | CTGTCCTCAGAGGCTTGTTTGAA | 2575 | 2604 | 2826 | 2849 |
|  | AMPL1678948 | TGGTGAGCAGATGACACTCTCA | ACTGCATTTTCCTGTTATCAGTTGGT | 2794 | 2816 | 3024 | 3050 |
|  | AMPL1678949 | GGCTGCAGCATTTCATGATGAA | GCTCTGAATTGACTGAAGATTCAAGGTATT | 2992 | 3014 | 3233 | 3263 |
|  | AMPL1678950 | CTGGATCATTGAAGAAGAGTGTGAATTAGA | TGAAAAATTCTTTAATGGTTGTTGCGTTGA | 3190 | 3220 | 3404 | 3434 |
|  | AMPL1678951 | CTGCTGGTCTGATGTCAACCAT | CAAGCAGTGGTTAGAATATTTGGTTTGT | 3372 | 3394 | 3593 | 3621 |
|  | AMPL1678952 | CTCGTCAGAATTCTCTGTTACATGGT | CCCTGGTTTGACAAAATGCTCTT | 3557 | 3583 | 3805 | 3828 |
|  | AMPL1678953 | AGGAAATTAAGGAACAAATTTGTGACACAG | TTTTGATTCCACAATGATATCAGAGGCATA | 3765 | 3795 | 4000 | 4030 |
|  | AMPL1678954 | AGAGGAATAAGGAGCAGGTTTGTG | TATCCAAACACCAATTGGTTCAACTCT | 3966 | 3990 | 4213 | 4240 |
|  | AMPL1678955 | ATACCATCAAGAATCAGCACCCAAA | TGAAGATATGCAATTGCAAAGCATTTCA | 4178 | 4203 | 4422 | 4450 |
|  | AMPL1678956 | ATAGATGATTTGGCCCAAACTAGGAAG | GGCTTGGCTAACATTGAGTTTACC | 4385 | 4412 | 4633 | 4657 |
|  | AMPL1678957 | CACATTAGGGCTGCAAAAGCTT | GGATTGCATCAGGGAAATTATGTTTAATGT | 4601 | 4623 | 4844 | 4874 |
|  | AMPL1678958 | AATCAGACCCTAGATGACTTGGATGA | AGCCTTGTTTCTCTCCACAAAACTT | 4808 | 4834 | 5049 | 5074 |
|  | AMPL1678959 | TGTGGATTGGTTCAGTAGCAAGATT | ATGCCATTTGTGATATTATATGCTCTAGCT | 5014 | 5039 | 5258 | 5288 |
|  | AMPL1678960 | AGGGAATTCAAAAATGAAGCTCCGTA | ACTTTAGAATGGCTAAATCCATGGGTT | 5225 | 5251 | 5468 | 5495 |
|  | AMPL1678961 | GCCCATCTGTGACACAAGTCAC | CACACATTCCTTTACATGATTTAACAGTGT | 5436 | 5458 | 5672 | 5702 |
|  | AMPL1678962 | CAGGGAAGGTACTGAAAGCACTAA | GCACCATAGACTGGACTCTTATGGAT | 5638 | 5662 | 5881 | 5907 |
|  | AMPL1678963 | CACCCATACAACCTATGTACATCAACA | CTGTTTTCATGTCTACAATGCCATTAACC | 5844 | 5871 | 6087 | 6116 |
|  | AMPL1678964 | AAAGCATGTGTCAAGTCCAAATTTAGG | TTTGAGACAAGTGTTAAAGATGGTGGTAA | 6050 | 6077 | 6293 | 6322 |
|  | AMPL1678965 | GTACAAGATAAGTTTCACAACCTCCTGAA | CAATCCTTGTATGGGTTAATACCCACT | 6254 | 6283 | 6471 | 6498 |
|  | AMPL1678966 | GACAAAGTATACCAAACTCCATGCTACTAT | ACCATCTCTCATTGAATACAACATGATCTG | 6431 | 6461 | 6662 | 6692 |
|  | AMPL1678967 | TCTCGTGATGCAACTCCATAAACC | CAACTCCAAATGAATCTGCCATGATAC | 6628 | 6652 | 6875 | 6902 |
|  | AMPL1678968 | CCCTGGATAAAGAAATTGAGCCAGA | TGGAGGCATAGTTCCATTAAGTAGGA | 6840 | 6865 | 7087 | 7113 |
|  | AMPL1678969 | GATGAAAAACTTCAGCACATTCAAGC | TTGGTATGTCCAATATTCCAAATTAGTGTTCT | 7051 | 7077 | 7292 | 7324 |
| Panel 3 (112741) | AMPL1820745 | GGGTCTCCTAGAGAGCTTGG | GTACAACACACTTTAAGCGTCTGAAC | 9 | 29 | 191 | 217 |
|  | AMPL1820746 | CTGGCCGAAGGCAACTAGCAAT | AGAGGCATCTGTTACCAGATGTG | 159 | 181 | 407 | 430 |
|  | AMPL1820747 | ATTTTGCTGCAAAGCATCCCATT | CCAACTCACCTAGATAGAGGTAGTTAGC | 374 | 397 | 605 | 633 |
|  | AMPL1820748 | CGTAGGTAACAAGTGACACTATGGA | CACACTAGACAAGGAGAGAGTATCCA | 570 | 595 | 743 | 769 |
|  | AMPL1820749 | GAGTCTATCAAGGATTTAGTCAATGTTGC | CTTGCACTACATCTTTATTCTCTGGTTG | 704 | 733 | 883 | 911 |
|  | AMPL1820750 | TCCAATTTACTTGGACCAAACAGTTTTG | CCACAGTGGTTATTTTGTACTGTCTAGAAT | 845 | 873 | 1088 | 1118 |
|  | AMPL1820751 | TAAGCTTATAAGATTGGACACAGCTTCATG | CTTCCTGCAGTTCCTTGGTTGA | 1048 | 1078 | 1223 | 1245 |
|  | AMPL1820752 | TAAGGTGTGGCTTTCATTTCCAAGT | GGTACTGAAAGAGGTGTCCAAACA | 1188 | 1213 | 1437 | 1461 |
|  | AMPL1820753 | CGATTCGTCTGATCTAGGGCAA | GTTCCAGGCCCTTCTGCTATATC | 1405 | 1427 | 1654 | 1677 |
|  | AMPL1820754 | GACTGCTTCAACTAAATACAAATGGACAC | GCCCAATCAAAGTACCCATATTTGTCTATA | 1615 | 1644 | 1854 | 1884 |
|  | AMPL1820755 | GTTATGTCAGACAACACTACACCTTCA | CTCCAACTGAGTATCATGAGTACAGTTG | 1817 | 1844 | 2058 | 2086 |
|  | AMPL1820756 | GTACTTTCAATAGAGGAAGGCTTAGGATG | ACTTCAACTTGAAATAAACCCAATTGGTG | 2019 | 2048 | 2188 | 2217 |
|  | AMPL1820757 | CCATATTCCTTCTCTACTTGGATGAGGA | TCATTTGGTGATATGGACTGTTTGCA | 2150 | 2178 | 2398 | 2424 |
|  | AMPL1820758 | AGATCCATTGTGCATTGAGGACA | GCCATGACCTTCTTTTGGAAATGT | 2365 | 2388 | 2614 | 2638 |
|  | AMPL1820759 | TCATGATTTTAACAATGGAGACACATGGA | CTGTCCTCAGAGGCTTGTTTGAA | 2575 | 2604 | 2826 | 2849 |
|  | AMPL1820760 | TGGTGAGCAGATGACACTCTCA | ACTGCATTTTCCTGTTATCAGTTGGT | 2794 | 2816 | 3024 | 3050 |
|  | AMPL1820761 | GGCTGCAGCATTTCATGATGAA | GCTCTGAATTGACTGAAGATTCAAGGTATT | 2992 | 3014 | 3233 | 3263 |
|  | AMPL1820762 | CTGGATCATTGAAGAAGAGTGTGAATTAGA | TGAAAAATTCTTTAATGGTTGTTGCGTTGA | 3190 | 3220 | 3404 | 3434 |
|  | AMPL1820763 | CTGCTGGTCTGATGTCAACCAT | CAAGCAGTGGTTAGAATATTTGGTTTGT | 3372 | 3394 | 3593 | 3621 |
|  | AMPL1820764 | CTCGTCAGAATTCTCTGTTACATGGT | CCCTGGTTTGACAAAATGCTCTT | 3557 | 3583 | 3805 | 3828 |
|  | AMPL1820765 | AGGAAATTAAGGAACAAATTTGTGACACAG | TTTGATTCCACAATGATATCAGAGGCAT | 3765 | 3795 | 4001 | 4029 |
|  | AMPL1820766 | GAGGAATAAGGAGCATGTTTGTGC | TATCCAAACACCAATTGGTTCAACTCT | 3967 | 3991 | 4213 | 4240 |
|  | AMPL1820767 | ATACCATCAAGAATCAGCACCCAAA | ACTGAAGATATGCAATTGCAAAGCATTT | 4178 | 4203 | 4424 | 4452 |
|  | AMPL1820768 | TAGATGATTTAGGCCAAACTAGGAAGGA | GGCTTGGCTAACATTGAGTTTACC | 4386 | 4414 | 4633 | 4657 |
|  | AMPL1820769 | CACATTAGGGCTGCAAAAGCTT | GGATTGCATCAGGGAAATTATGTTTAATGT | 4601 | 4623 | 4844 | 4874 |
|  | AMPL1820770 | AATCAGACCCTAGATGACTTGGATGA | AGCCTTGTTTCTCTCCACAAAACTT | 4808 | 4834 | 5049 | 5074 |
|  | AMPL1820771 | TGTGGATTGGTTCAGTAGCAAGATT | ATGCCATTTGTGATATTATATGCTCTAGCT | 5014 | 5039 | 5258 | 5288 |
|  | AMPL1820772 | AAGGGAATTCAAAAATGAAGCTCCCTA | ACTTTAGAATGGCTAAATCCATGGGTT | 5224 | 5251 | 5468 | 5495 |
|  | AMPL1820773 | GCCCATCTGTGACACAAGTCAC | CACACATTCCTTTACATGATTTAACAGTGT | 5436 | 5458 | 5672 | 5702 |
|  | AMPL1820774 | CAGGGAAGGTACTGAAAGCACTAA | GCACCATAGACTGGACTCTTATGGAT | 5638 | 5662 | 5881 | 5907 |
|  | AMPL1820775 | CACCCATACAACCTATGTACATCAACA | CTGTTTTCATGTCTACAATGCCATTAACC | 5844 | 5871 | 6087 | 6116 |
|  | AMPL1820776 | AAAGCATGTGTCAAGTCCAAATTTAGG | TTTGAGACAAGTGTTAAAGATGGTGGTAA | 6050 | 6077 | 6293 | 6322 |
|  | AMPL1820777 | GTACAAGATAAGTTTCACAACCTCCTGAA | CAATCCTTGTATGGGTTAATACCCACT | 6254 | 6283 | 6471 | 6498 |
|  | AMPL1820778 | GACAAAGTATACCAAACTCCATGCTACTAT | ACCATCTCTCATTGAATACAACATGATCTG | 6431 | 6461 | 6662 | 6692 |
|  | AMPL1820779 | TCTCGTGATGCAACTCCATAAACC | CAACTCCAAATGAATCTGCCATGATAC | 6628 | 6652 | 6875 | 6902 |
|  | AMPL1820780 | CCCTGGATAAAGAAATTGAGCCAGA | TGGAGGCATAGTTCCATTAAGTAGGA | 6840 | 6865 | 7087 | 7113 |
|  | AMPL1820781 | GATGAAAAACTTCAGCACATTCAAGC | TTGGTATGTCCAATATTCCAAATTAGTGTTCT | 7051 | 7077 | 7292 | 7324 |

Three custom Ion AmpliSeq Panels were generated for use with the Ion Torrent S5 System. Primer sets for Panels 2 and 3 were designed based on updated sequences of the Australian HPeV3 samples. Panel 1 (not shown) was based on the Yamagata 2011 genome as detailed previously ^1^.

1 Nelson, T. M. *et al.* An outbreak of severe infections among Australian infants caused by a novel recombinant strain of human parechovirus type 3. *Sci Rep* **7**, 44423, doi:10.1038/srep44423 (2017).
